# Identification of cold tolerance QTLs at the bud burst stage in 211 rice landraces by GWAS

**DOI:** 10.1186/s12870-021-03317-7

**Published:** 2021-11-20

**Authors:** Caijing Li, Jindong Liu, Jianxin Bian, Tao Jin, Baoli Zou, Shilei Liu, Xiangyu Zhang, Peng Wang, Jingai Tan, Guangliang Wu, Qin Chen, Yanning Wang, Qi Zhong, Shiying Huang, Mengmeng Yang, Tao Huang, Haohua He, Jianmin Bian

**Affiliations:** 1 Key Laboratory of Crop Physiology, Ecology and Genetic Breeding, Ministry of Education, Nanchang, 330045 Jiangxi Province China; 2 Key Laboratory of Crop Physiology, Ecology and Genetic Breeding, Nanchang, 330045 Jiangxi Province China; 3 Agricultural Genomics Institute in Shenzhen, Chinese Academy of Agricultural Sciences, Shenzhen, 518000 Guangdong Province China; 4 Peking University Institute of Advanced Agricultural Sciences, Weifang, 261325 Shandong Province China

**Keywords:** Cold tolerance, Rice landraces, GWAS, Seedling survival rate (SR), QTL

## Abstract

**Background:**

Rice is a crop that is very sensitive to low temperature, and its morphological development and production are greatly affected by low temperature. Therefore, understanding the genetic basis of cold tolerance in rice is of great significance for mining favorable genes and cultivating excellent rice varieties. However, there have been limited studies focusing on cold tolerance at the bud burst stage; therefore, considerable attention should be given to the genetic basis of cold tolerance at this stage.

**Results:**

In this study, a natural population consisting of 211 rice landraces collected from 15 provinces in China and other countries was used for the first time to evaluate cold tolerance at the bud burst stage. Population structure analysis showed that this population was divided into two groups and was rich in genetic diversity. Our evaluation results confirmed that *japonica* rice was more tolerant to cold at the bud burst stage than *indica* rice. A genome-wide association study (GWAS) was performed with the phenotypic data of 211 rice landraces and a 36,727 SNP dataset under a mixed linear model. Twelve QTLs (*P* < 0.0001) were identified for the seedling survival rate (SR) after treatment at 4 °C, in which there were five QTLs (*qSR2–2*, *qSR3–1*, *qSR3–2*, *qSR3–3* and *qSR9*) that were colocalized with those from previous studies and seven QTLs (*qSR2–1*, *qSR3–4*, *qSR3–5*, *qSR3–6*, *qSR3–7*, *qSR4* and *qSR7*) that were reported for the first time. Among these QTLs, *qSR9*, harboring the most significant SNP, explained the most phenotypic variation. Through bioinformatics analysis, five genes (*LOC_Os09g12440*, *LOC_Os09g12470*, *LOC_Os09g12520*, *LOC_Os09g12580* and *LOC_Os09g12720*) were identified as candidates for *qSR9*.

**Conclusion:**

This natural population consisting of 211 rice landraces combined with high-density SNPs will serve as a better choice for identifying rice QTLs/genes in the future, and the detected QTLs associated with cold tolerance at the bud burst stage in rice will be conducive to further mining favorable genes and breeding rice varieties under cold stress.

**Supplementary Information:**

The online version contains supplementary material available at 10.1186/s12870-021-03317-7.

## Background

Originating in tropical and subtropical areas, rice is one of the main staple foods worldwide. Low temperature has a large impact on rice growth and cultivation. More than 15 million hectares of rice cultivated area worldwide have been affected by low temperatures. Rice is cultivated in a large area in China, ranging from 53°27′ to 18° 90′ north latitude, especially in the provinces of the Yangtze River basin, and from 1951 to 1980, rice in the Yangtze River basin suffered from severe cold injury, with losses of 5 to 10 billion kilograms of rice every year [1]. Early rice in the Yangtze River basin in China is often affected by cold injury at the bud burst stage, resulting in a low germination rate and failure to emerge. Therefore, it is necessary to study cold tolerance in rice at the bud burst stage.

Cold tolerance is a complex quantitative trait that is often controlled by multiple genes and the environment, and researchers often use biparental populations to look for QTLs associated with cold tolerance. Researchers have found more than 250 QTLs in biparental populations using traditional QTL mapping methods during various stages of rice growth [2]. Among more than 250 QTLs, many genes throughout a variety of rice growth stages have been isolated. At the germination stage, *qLTG3–1* was the first gene to be linked to germination at low temperatures [3]. During the seedling stage of rice, many QTLs/genes related to cold tolerance have been isolated, including *qCTS12* [4], *qCTS4* [5], *qCtss11* [6], *qSCT1* and *qSCT11* [7], *qLOP2* and *qPSR2–1* [8], *COLD1* [9] and *qCTS-9* [10]. *qCTS12* was the first cold tolerance gene identified at the seedling stage. *COLD1* is another important gene related to cold tolerance at the seedling stage, and it is also the first cold-tolerance gene identified to be involved in signal transduction. At the booting stage of rice, several genes have been isolated, including *Ctb1* [11], *qCT8* [12], *qCTB7* [13], *qCTB3* [14] and *qCT-3-2* [15]. *Ctb1* is the first gene to be linked to cold tolerance at the booting stage in rice. Although biparental mapping populations have played a great role in traditional gene mapping, the construction of biparental populations entails a major investment in time and has therefore limited the number of genes identified to date [16].

To identify new genes associated with target traits, the exploration of QTLs/genes through natural populations with weak genetic relationships using GWAS has become one of the most popular methods. This method eliminates the need to construct a mapping population and can simultaneously analyze multiple alleles using recombination information from the long-term evolution of natural populations. In recent years, some studies have applied this method to study rice cold tolerance. Seventeen QTLs related to rice germination at low temperatures were detected using 63 Japanese varieties [17]. Fifty-one QTLs were mapped by GWAS with the a population of 174 rice accessions from China [18]. One hundred and thirty-two QTLs for both rice natural chilling and cold shock stresses were identified in 527 rice varieties [19]. Sixty-seven QTLs were mapped for cold stress at the seedling stage of rice, and fifty-six QTLs were newly discovered [20]. Forty-two QTLs were found to be associated with cold tolerance at the rice seedling stage, twenty of which have not been mentioned in previous reports [21]. Thirty-one QTLs related to the low-temperature germination of rice seeds at the rice germination stage were detected using 200 *japonica* rice varieties [22]. Forty-seven QTLs were identified for cold tolerance at the bud burst stage using 249 *indica* rice accessions [23]. Twenty-six QTLs were found to be related to cold tolerance at the rice seedling stage by using a core collection of landraces of rice selected from 2262 accessions of Ting’s collection [24]. In addition, thirty-one distinct QTL regions were identified for low-temperature germination in a panel of 257 rice accessions from all areas of the world [25]. However, there are still few studies using GWAS to explore cold tolerance in rice at the bud burst stage. To understand the genetic mechanism of cold tolerance in rice at an early stage, it is necessary to search for QTLs related to cold tolerance in rice at the bud burst stage.

In this study, we selected 211 rice landraces from different regions to form a natural population and performed high-throughput sequencing using a microarray. The 211 rice landraces were mainly composed of *indica* and *japonica* rice, which provided abundant genetic diversity for studying the cold tolerance of rice. We treated plants from the natural population with low temperature (4 °C) and then recovered them at room temperature. A total of 12 QTLs, as well as 5 candidate genes for *qSR9,* related to cold tolerance were identified by our GWAS of SR, which provided valuable genetic resources for cold tolerance research in rice and laid a solid foundation for breeding cold tolerant rice varieties.

## Results

### Cold tolerance of the 211 rice varieties

**Fig. 1:**
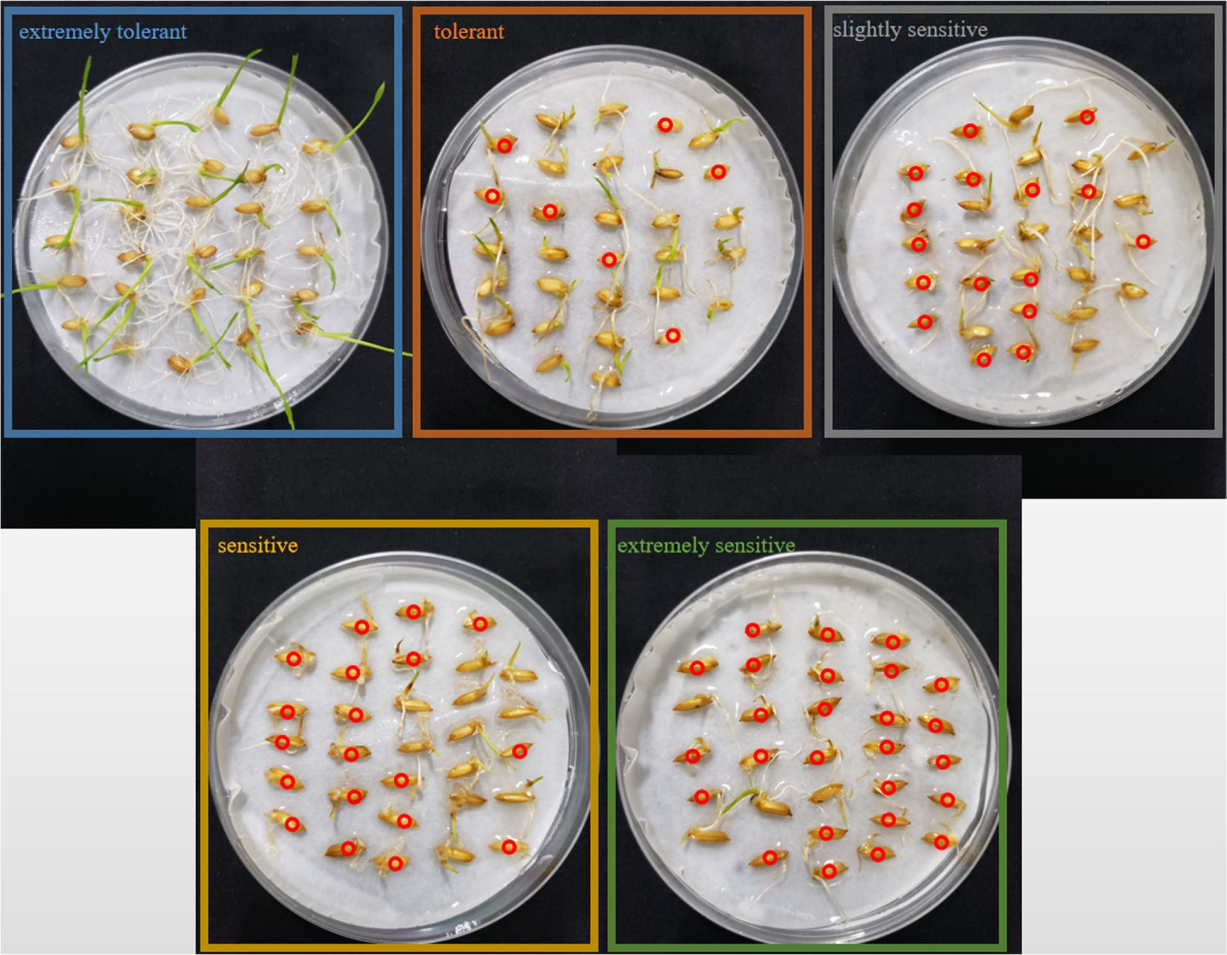
Variations in cold tolerance at bud burst stage in 211 rice landraces. The five colored frame lines represent the five levels of tolerance to cold stress, and the small red circles represent the dead individuals after cold stress

**Fig. 2:**
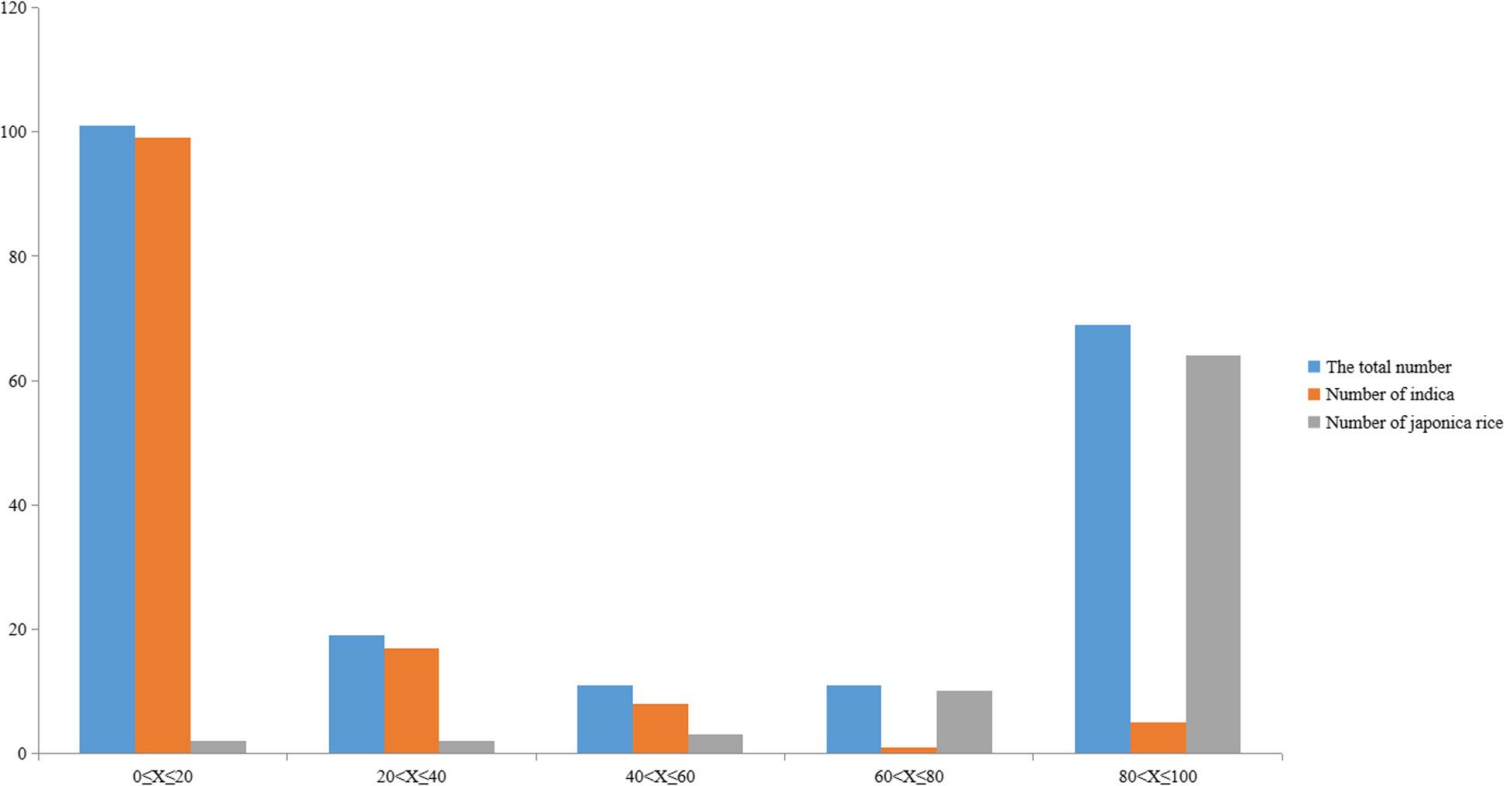
Histogram of SR. The blue area represents the total number of rice samples, the orange area represents *indica* rice, and the gray area represents *japonica* rice; the horizontal axis represents the cold tolerance level

In our study, SR was used to evaluate cold tolerance at the bud burst stage (Table S2). Due to the abundant landrace germplasm resources, the phenotypes of rice varieties within the natural population varied greatly after 4 °C treatment **(**Fig. 1**)**. We classified cold tolerance into five levels of SR: extremely sensitive (0 ≤ X ≤ 20), sensitive (20 < X ≤ 40), slightly sensitive (40 < X ≤ 60), tolerant (60 < X ≤ 80), and extremely tolerant (80 < X ≤ 100) **(**Table 1**;** Fig. 2**)**. Of the 101 rice varieties that were extremely sensitive to low temperature, 99 were *indica* and 2 were *japonica*. Correspondingly, of the 69 rice varieties that were extremely tolerant to low temperature, 5 were *indica* and 64 were *japonica*. According to SPSS software 26.0 analysis results, the SR was significantly correlated with the *indica* and *japonica* types, and the correlation coefficient was 0.851 **(**Table 2**)**, suggesting that *japonica* was more cold tolerant than *indica* at the bud burst stage*.* On the other hand, we found that the cold tolerance of rice was significantly related to its geographical distribution, and the correlation coefficient was 0.714 **(**Table 2**)**. The higher the latitude was, the stronger the cold tolerance of rice, which may be due to the significant correlation between *indica* or *japonica* types and latitude **(**Table 2**)**.

**Table 1:** SR (seedling survival rate) after cold treatment at the bud burst stage

SR (%)	The total number	Number of *indica*	Number of *japonica*
0 ≤ X ≤ 20	101	99	2
20 < X ≤ 40	19	17	2
40 < X ≤ 60	11	8	3
60 < X ≤ 80	11	1	10
80 < X ≤ 100	69	5	64

**Table 2:** Correlations between SR, latitude and *indica* or *japonica* type

Traits	SR	Latitude	*Indica* or *japonica* type
SR		0.714^a^	0.851^a^
Latitude			0.910^a^
*Indica* or *japonica* type			

^a^Indicates significance at the 1% level

### Population structure and relative kinship

**Fig. 3:**
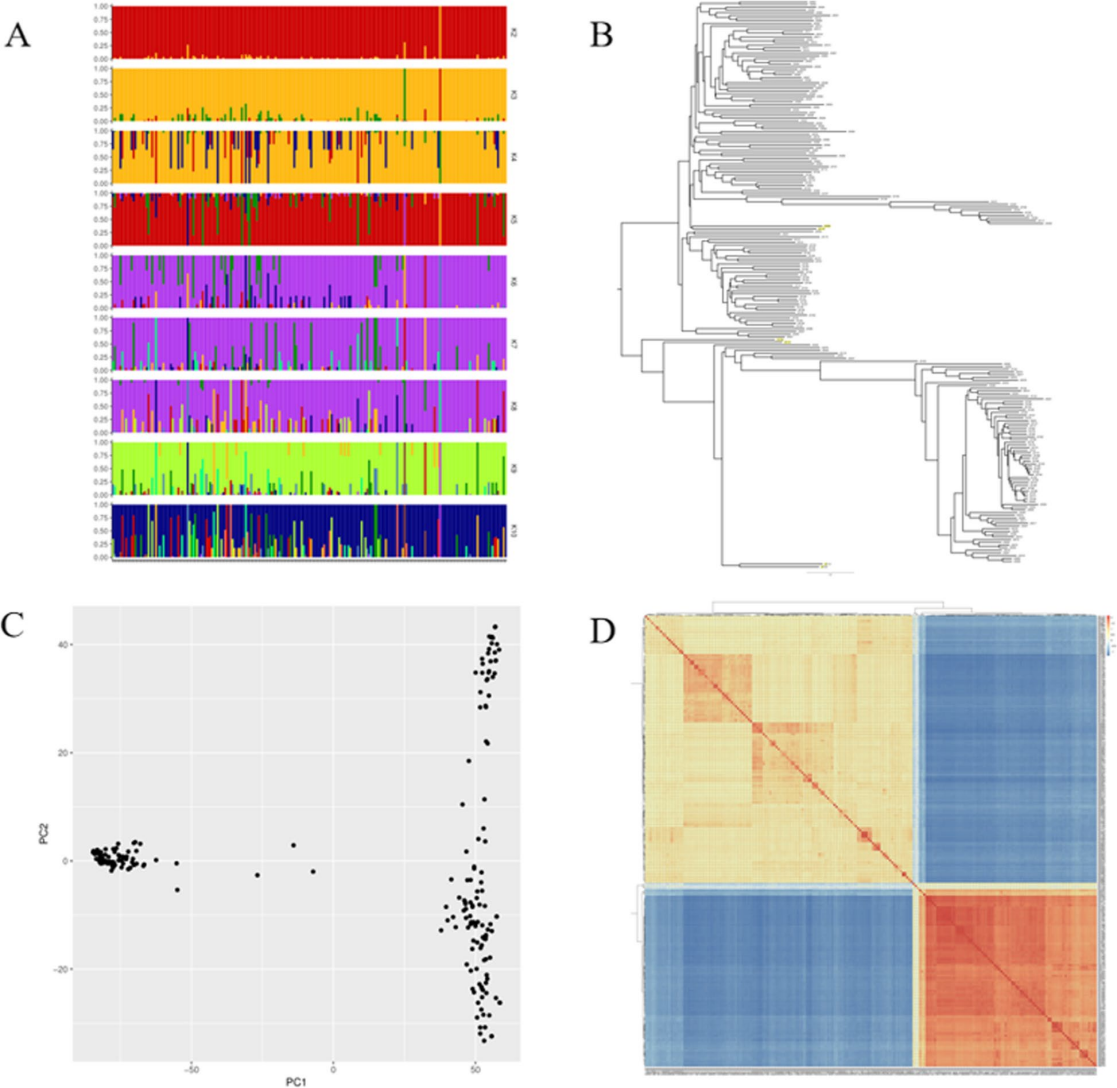
Population structure of 211 landraces **A.** Subgroups (K = 2) inferred using structure software; yellow and red represent subgroups I and II, respectively; **B.** NJ tree based on Nei’s genetic distances; **C.** PCA of 211 rice varieties; **D.** Pairwise relative kinship analysis of the rice panel

STRUCTURE, a neighbor-joining (NJ) tree analysis, a principal component analysis (PCA) and kinship were used to analyze the population structure of the natural population based on 36,727 SNPs **(**Fig. 3**)**. According to the STRUCTURE analysis, the log likelihood increased gradually from K = 1 to K = 10. The maximum ad hoc measure 1 K was observed for K = 2, which indicated that the entire population could be divided into two subgroups **(**Fig. 3A**)**. The 211 rice varieties could be divided into 2 subgroups by the NJ tree **(**Fig. 3B**)** and the two principal components from this panel **(**Fig. 3C**)**. In addition, in the relationship analysis, we found that there were two major subgroups in the 211 rice varieties **(**Fig. 3D**)**, suggesting that landraces population germplasm resources had abundant genetic diversity, which was beneficial for performing GWAS.

### GWAS analysis for cold tolerance at the bud burst stage

**Fig. 4:**
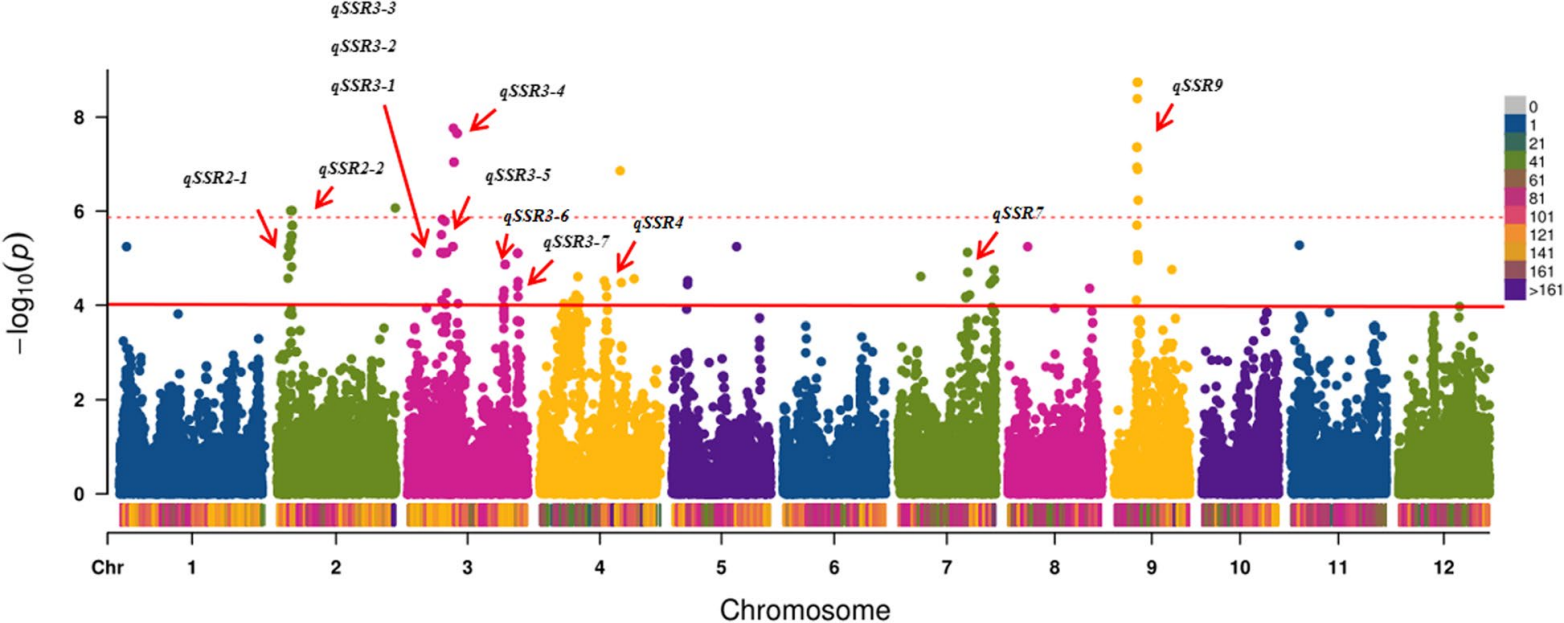
Manhattan plots of GWAS for SR. The dotted line represents the Bonferroni correction and the solid line represents *P* = 0.0001

**Fig. 5:**
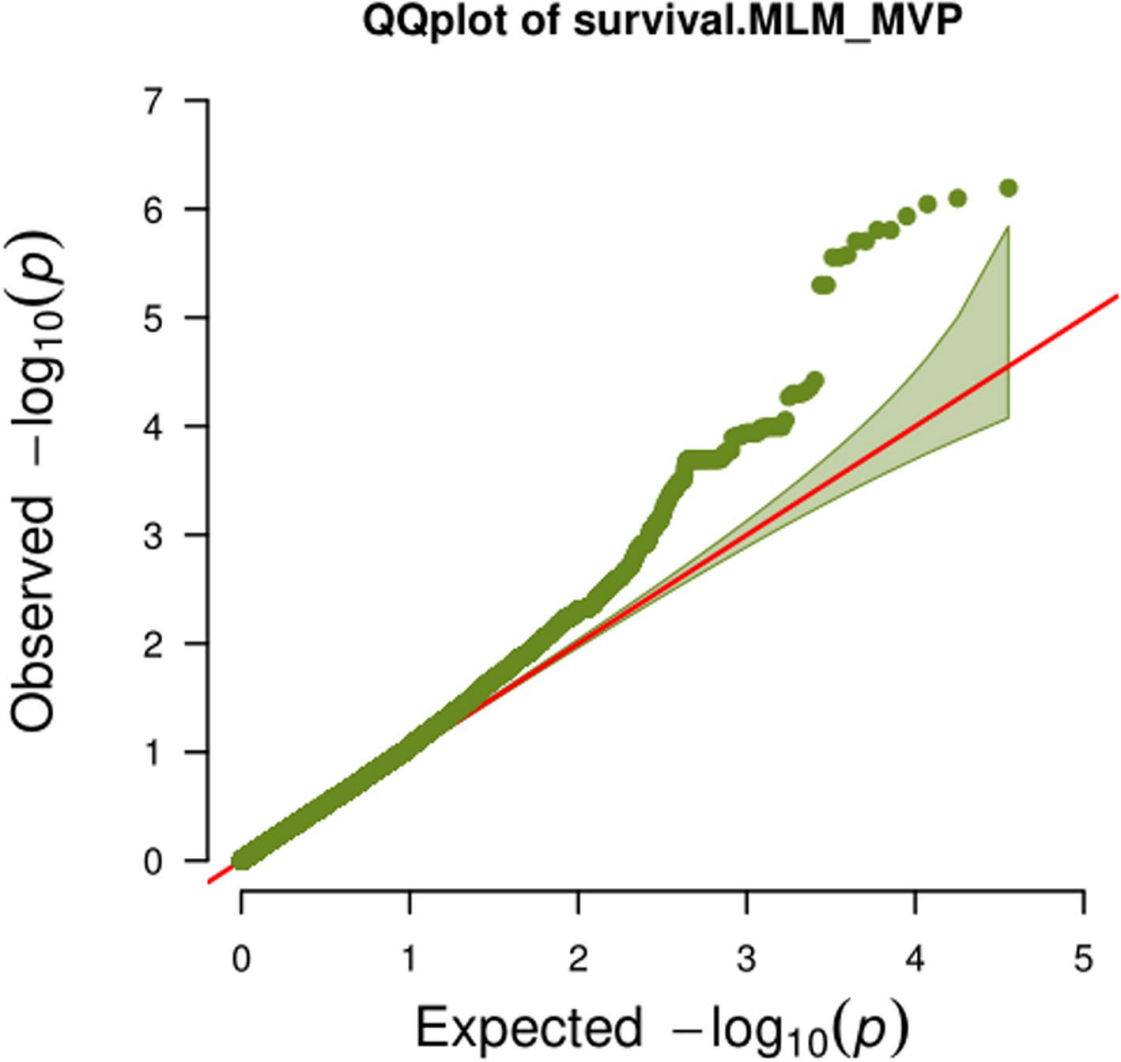
Quantile–quantile (Q–Q) plot of GWAS for SR

Based on genotype and phenotype data, an association analysis was performed under a mixed linear model using the PCA and KINSHIP results as co-variables. A total of 12 QTLs (*qSR2–1*, *qSR2–2*, *qSR3–1*, *qSR3–2*, *qSR3–3*, *qSR3–4*, *qSR3–5*, *qSR3–6*, *qSR3–7*, *qSR4*, *qSR7* and *qSR9*) were identified in SR with well-fitted quantile–quantile (Q–Q) plots (*p <* 0.0001) **(**Fig. 4 and Fig. 5; Table 3). A QTL, *qSR2–2,* on chromosome 2 overlapped with *OsWRKY71*. Another QTL on chromosome 2, *qSR2–1*, has not been previously reported. On chromosome 3, there were three QTLs overlapping with those from previous studies, namely, *qSR3–1*, *qSR3–2* and *qSR3–3*, and the other four QTLs on chromosome 3 were newly discovered and have not been reported by previous studies. The remaining two QTLs on chromosomes 4 (*qSR4*) and 7 (*qSR7*) were also newly discovered in this study. In addition, we also found a QTL on chromosome 9, *qSR9*, which harbored the most significant SNP and explained most of the phenotypic variation. This QTL is located at approximately 7.1 Mb on chromosome 9 and colocalizes with *clr9*.

**Table 3:** Summary of the significant SNPs detected by GWAS and the overlapping QTLs/genes reported previously

QTL ID	Trait	Chr.	Peak SNPs	*P* value	R^2^	Previous QTL/genes
*qSR2–1*	seedling survival rate	2	3,379,509	8.99E-06	0.076575784	
*qSR2–2*	seedling survival rate	2	4,435,145	9.70E-07	0.085670420	*OsWRKY71 *[26]
*qSR3–1*	seedling survival rate	3	10,230,171	3.16E-06	0.071228227	*qLVG3 *[27]
*qSR3–2*	seedling survival rate	3	10,552,889	1.48E-06	0.068345879	*qLVG3 *[27]
*qSR3–3*	seedling survival rate	3	11,316,517	1.64E-06	0.057976301	*qLVG3 *[27]
						*OsMYB2 *[28]
						*OsCIPK03* [29]
*qSR3–4*	seedling survival rate	3	13,860,165	1.72E-08	0.111646171	
*qSR3–5*	seedling survival rate	3	15,042,189	2.19E-08	0.094111949	
*qSR3–6*	seedling survival rate	3	29,271,308	4.89E-05	0.038274826	
*qSR3–7*	seedling survival rate	3	33,444,741	7.77E-06	0.048865413	
*qSR4*	seedling survival rate	4	11,487,556	2.46E-05	0.03888723	
*qSR7*	seedling survival rate	7	29,111,646	1.77E-05	0.039475464	
*qSR9*	seedling survival rate	9	7,106,185	4.07E-09	0.136902573	*clr9 *[30]

### Candidate gene analysis

**Fig. 6:**
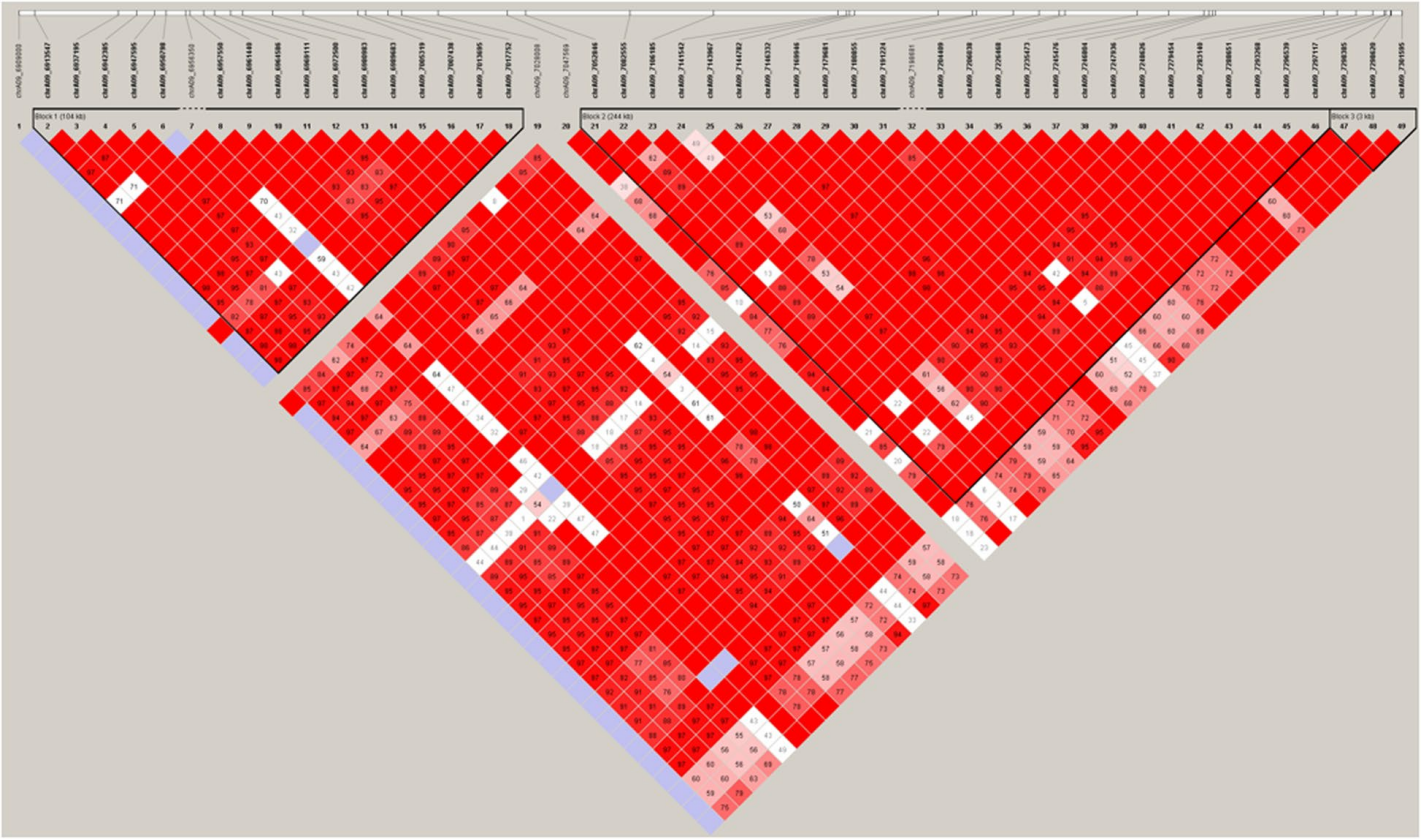
LD heatmap around the peak on chromosome 9

**Fig. 7:**
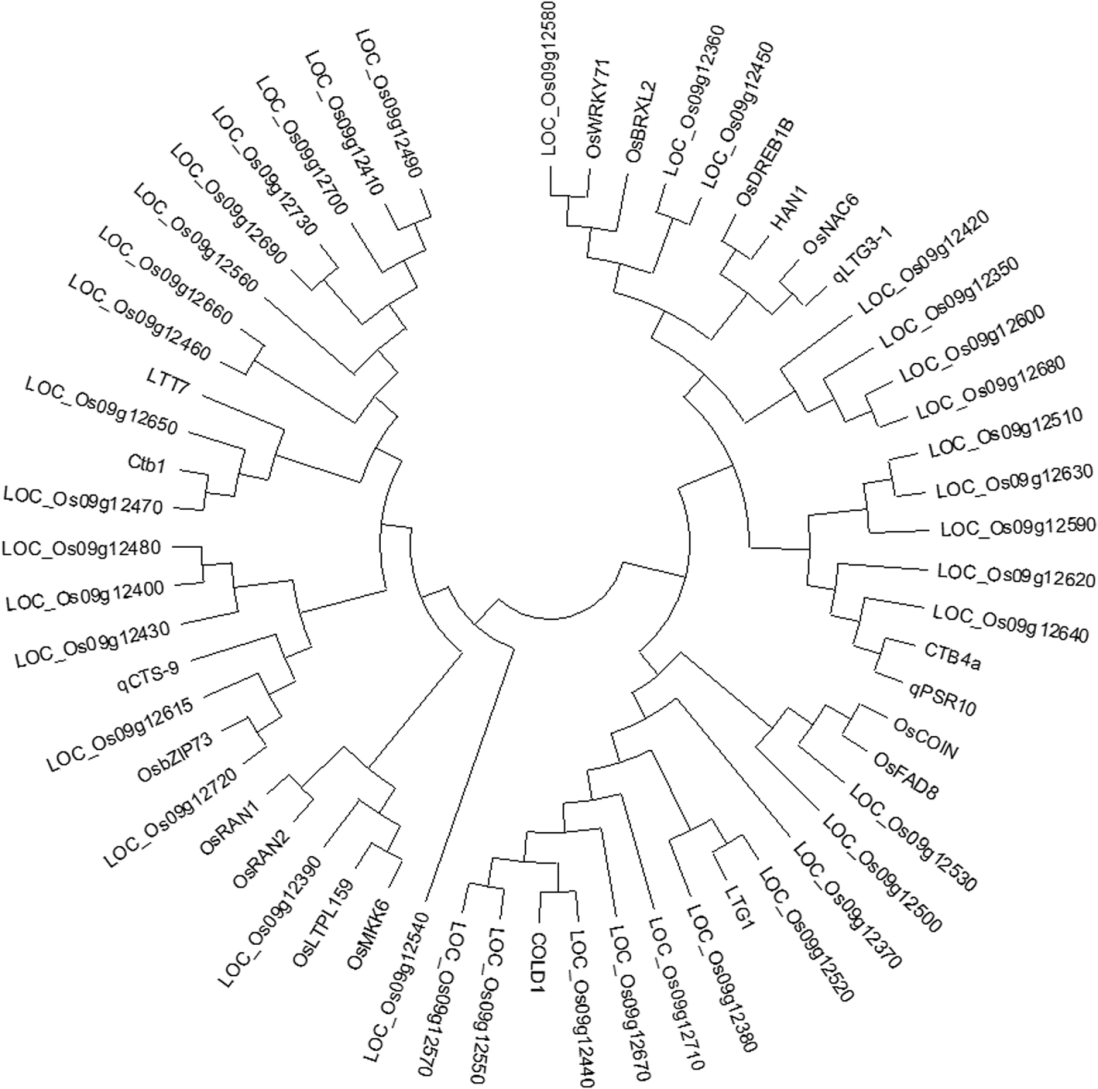
Homology analysis among of 59 genes (39 putative genes and 20 reference genes)

Among these QTLs, we conducted further candidate genetic analysis of *qSR9*. According to the LD decay analysis, a 244-kb region was identified as the candidate region **(**Fig. 6**).** There were 39 genes in this region, including 3 hypothetical proteins, 4 transposon proteins, 7 retrotransposon proteins and 16 functionally annotated genes (Table S3). To identify possible candidate genes, we analyzed the homology between these 39 genes and 20 characterized cold-tolerance genes. *LOC_Os09g12440*, *LOC_Os09g12470*, *LOC_Os09g12520*, *LOC_Os09g12580* and *LOC_Os09g12720* were found to have a high degree of homology with *COLD1*, *Ctb1*, *LTG1*, *OsWRKY71* and *OsbZIP73* **(**Fig. 7**;** Table 4**)**.

**Table 4:** Candidate genes in the *qSR9* region

QTL	Putative genes	Putative protein functions	Reference genes
*qSR9*	***LOC_Os09g12440***	retrotransposon protein, putative, unclassified, expressed	*COLD1*
	***LOC_Os09g12470***	hypothetical protein	*CTB1*
	***LOC_Os09g12520***	hypothetical protein	*LTG1 *[31]
	***LOC_Os09g12580***	expressed protein	*OsWRKY71*
	***LOC_Os09g12720***	zinc finger, C3HC4 type domain containing protein, expressed	*OsbZIP73 *[32]

## Discussion

### Population structure and phenotypic assessment of a natural population

For this study, GWAS was used to reveal the complicated genetic variations related to cold tolerance. However, population structure is an important factor affecting the results of GWAS and increases the false positive rate. In this study, a natural population consisting of 211 rice landraces (130 *indica* rice and 81 *japonica* rice) was used to assess cold tolerance in rice at the bud burst stage. Most of the rice varieties come from 15 provinces in China, with three from Japan and one from the Philippines. The geographical regions span from 15° to 48° north latitude, including temperate zones, tropics and subtropics. This natural population is newly constructed and has rich genetic diversity. Population structure analysis divided the natural population into two groups. Subsequently, the results of PCA and NJ tree construction support the low relatedness identified by the kinship analysis **(**Fig. 3**)**, which makes these data suitable for GWAS.

We used the SR as the indicator to evaluate the cold tolerance of natural populations. The results show that the SR ranged from 0 to 100% (Table S2); *indica* rice is extremely sensitive to temperature, and its SR is low after cold treatment. Some *indica* rice varieties even died in the recovery time. *Japonica* rice is cold tolerant, and the SR of most *japonica* rice varieties is above 90%. This result showed that *japonica* rice was more tolerant to cold than *indica* rice at the bud burst stage.

### Identification of QTLs/candidate genes controlling cold tolerance at the bud burst stage

In this study, we found 12 QTLs for SR after cold treatment. Among these QTLs, seven (*qSR2–1*, *qSR3–4*, *qSR3–5*, *qSR3–6*, *qSR3–7*, *qSR4* and *qSR7*) were reported for the first time, and the other five QTLs (*qSR2–2*, *qSR3–1*, *qSR3–2*, *qSR3–3* and *qSR9*) were colocalized with QTLs from previous studies. The peak SNP for *qSR2–2* was located at 4.4 Mb on chromosome 2. This region overlapped with *OsWRKY71*, which is a transcriptional suppressor that encodes GA signaling in aleurone cells and cold tolerance. *qSR3–1*, *qSR3–2* and *qSR3–3* were colocalized with *qLVG3*, a QTL for low-temperature vigor during germination. In the *qLVG3* region, there were two characterized cold tolerance genes (*OsMYB2* and *OsCIPK03*). *OsMYB2* is a MYB transcription factor and plays a regulatory role in the tolerance of rice to salt, cold injury and dehydration stress, while *OsCIPK03* is a calcineurin B-like protein-interacting protein kinase. The overexpression of *OsCIPK03* in transgenic plants significantly improved their tolerance to cold stress. The QTL *qSR9* explained the largest phenotypic variation in our study, overlapping with *clr9*, which is a QTL associated with the culm length growth rate under cold stress; however, knowledge about the candidate genes underlying *qSR9* is still lacking.

The abundant SNP dataset for our natural population obtained through a chip strategy makes it feasible to locate *qSR9* to a small genomic region. The candidate gene analysis showed that there were 39 candidate genes underlying *qSR9*. Among these candidate genes, five genes (*LOC_Os09g12440*, *LOC_Os09g12470*, *LOC_Os09g12520*, *LOC_Os09g12580* and *LOC_Os09g12720*) might be the target genes because these five candidate genes are homologous to the characterized cold tolerance genes *COLD1*, *Ctb1*, *LTG1*, *OsWRKY71* and *OsbZIP73,* respectively **(**Fig. 7**)***.* For these characterized cold tolerance genes, *COLD1* encodes a G protein signal regulator and can interact with RGA1, the alpha subunit of the G protein, to sense low temperature, activate Ca^2+^ channels, and enhance the activity of the G protein GTP enzyme to enhance the cold tolerance of rice. *Ctb1* encodes an F-box protein, which interacts with the E3 ubiquitin ligase subunit SKP1 and is involved in cold tolerance at the booting stage. *LTG1* encodes a casein kinase, regulates the cold response in rice, affects auxin transport, synthesis and signal transduction, and positively regulates the low-temperature tolerance of rice during the vegetative growth period. *OsbZIP73*^Jap^ is upregulated by low temperature and the plant hormone abscisic acid (ABA), suggesting that *OsbZIP73* is involved in ABA-dependent low-temperature signaling pathways. However, other genes underlying *qSR9* cannot be ruled out, such as *LOC_Os09g12360*, *LOC_Os09g12390*, *LOC_Os09g12450*, *LOC_Os09g12615*, *LOC_Os09g12640* and *LOC_Os09g12650*. Although these genes are not homologous to the these characterized cold tolerance genes, they are very homologous to other characterized cold tolerance genes **(**Fig. 7**)**. Further studies, such as gene complementation analysis, are necessary to elucidate which allele is more favorable.

## Conclusions

In this study, a natural population consisting of 211 rice landraces was used to assess cold tolerance at the bud burst stage by using GWAS under a mixed linear model. Twelve QTLs were detected on chromosomes 2, 3, 4, 7, and 9, and five genes (*LOC_Os09g12440, LOC_Os09g12470, LOC_Os09g12520, LOC_Os09g12580 and LOC_Os09g12720*) might be the target genes for *qSR9* after candidate gene analysis. These QTLs/genes will be conducive to further mining favorable genetic resources and breeding rice varieties. The highlight of this study is the combination of previously unstudied rice landraces with the latest sequencing technology and the discovery of a number of new QTLs related to cold tolerance in rice that will assist in the breeding of new rice varieties with cold tolerance.

## Methods

### Plant material

A natural population of 211 rice varieties selected from International Rice Research Institute (https://www.irri.org/) was used to evaluate the cold tolerance of rice at the bud burst stage (Table S1). These varieties were mainly collected from 15 different provinces in China as well as from the Philippines and Japan. Their geographical range spans from 15° to 48° north latitude, including temperate, tropical and subtropical regions. Of the 211 rice varieties, 81 were classified as *japonica* rice, and 130 were classified as *indica* rice. The rice materials were collected in accordance with local laws without any conflict of interest. The population was developed in the experimental field at Jiangxi Agricultural University in Nanchang, Jiangxi Province, and Linwang, Hainan Province, for more than two generations.

### Cold tolerance evaluation at the bud burst stage

The tested seeds were placed in an oven at 45 °C for 48 h to break seed dormancy. The seeds were disinfected with sodium hypochlorite solution and rinsed with sterile water three times. Next, the seeds were soaked in water for approximately 48 h and allowed to germinate for 24 h. Thirty seeds with 5 mm coleoptile lengths were selected and placed in a petri dish with two sheets of filter paper. The petri dishes were placed in a growth incubator at 4 °C and treated in darkness for 10 days. After cold treatment, the petri dishes containing the seeds were placed in an incubator at 25 °C (14-h light/10-h dark) and recovered for 5 days. The seedling survival rate (SR) was calculated after 5 days of recovery of growth and used as the indicator of cold tolerance at the bud burst stage. Seedling survival rate (%) = number of surviving seeds/30 × 100. All experiments were repeated three times.

### GWAS mapping of rice cold tolerance QTLs/genes at the bud burst stage

Tassel 5.0 software was used for the GWAS of rice cold tolerance at the bud burst stage. Principal component analysis (PCA) and kinship analysis were performed using a 36,727 SNP genotype dataset. After filtering, standardized phenotype data, SNP data and principal component data were merged, and GWAS was performed with kinship data as covariables under a mixed linear model. Plotting was performed with the R package R**_**MVP. QTL regions were identified when 3 or more significant SNPs occurred within a 400 kb interval. Candidate gene analyses of *qSR9* were conducted with RAP-DB (https://rapdb.dna.affrc.go.jp/) and NCBI (https://www.ncbi.nlm.nih.gov/). The DNA sequences of the putative genes and cold tolerance genes were downloaded from NCBI and analyzed with MEGA-X software (https://www.megasoftware.net/).

### Statistical and genetic analyses

A correlation analysis of the SR, latitude and *indica* or *japonica* type was carried out with SPSS Statistics 26 software.

### Genomic DNA extraction, sequencing and genotyping

The CTAB method was used to extract DNA from approximately 100 mg of fresh young leaves, and the quantity and quality of DNA were measured using a Denovix DS-11 spectrophotometer. In addition, purity was determined by electrophoresis of the DNA for 1 h at 60 V in a 1% agarose gel. The 50 K rice gene SNP microarray ‘OsSNPNKs’ was used for genotyping. The SNPs on the microarrays were evenly distributed throughout the genome, with an average distance of < 1 kb between SNPs. Genotyping was based on Affymetrix AXIOM®2.0. The Target Prep Protocol QRC (P/N 702990) Kit was used for DNA amplification, DNA fragmentation, microarray hybridization, DNA binding single base extension, and signal amplification. Staining and scanning were performed using a GeneTitan® multichannel instrument [33].

## Supplementary Information

**Additional file 1: Table S1.** Geographical distribution and *indica* or *japonica* type of 211 rice varieties

**Additional file 2: Table S2**. SR of the 211 rice varieties

**Additional file 3: Table S3**. 39 candidate genes for *qSR9*

**Additional file 4: Table S4**. The SNPs data used for performed GWAS

## Data Availability

The sequencing data generated in this study were deposited in the databases (https://snp-seek.irri.org/). In addition, we have also sorted out a genotype data Table for GWAS based on the sequencing data, please refer to Table S4 for details.

## Abbreviations

SR: Seedling survival rate
QTL: Quantitative trait loci
GWAS: Genome-wide association study
SNP: Single nucleotide polymorphism
PCA: Principal component analysis
NJ: Neighbor-joining
